# Vitreous Humor Proteome: Targeting Oxidative Stress, Inflammation, and Neurodegeneration in Vitreoretinal Diseases

**DOI:** 10.3390/antiox11030505

**Published:** 2022-03-06

**Authors:** Fátima Milhano Santos, Joana Mesquita, João Paulo Castro-de-Sousa, Sergio Ciordia, Alberto Paradela, Cândida Teixeira Tomaz

**Affiliations:** 1 CICS-UBI—Centro de Investigação em Ciências da Saúde, Universidade da Beira Interior, 6201-001 Covilhã, Portugal; d1393@ubi.pt or jpcastrosousa@netcabo.pt (J.P.C.-d.-S.); 2 Unidad de Proteomica, Centro Nacional de Biotecnología, CSIC, Campus de Cantoblanco, 28049 Madrid, Spain; sciordia@cnb.csic.es (S.C.); alberto.paradela@cnb.csic.es (A.P.); 3 C4-UBI, Cloud Computing Competence Centre, University of Beira Interior, 6200-501 Covilhã, Portugal; 4 Department of Ophthalmology, Centro Hospitalar de Leiria, 2410-197 Leiria, Portugal; 5 Chemistry Department, Faculty of Sciences, University of Beira Interior, 6201-001 Covilhã, Portugal

**Keywords:** age-related macular degeneration, inflammation, neurodegeneration, oxidative stress, proliferative diabetic retinopathy, proliferative vitreoretinopathy, vitreous proteomics

## Abstract

Oxidative stress is defined as an unbalance between pro-oxidants and antioxidants, as evidenced by an increase in reactive oxygen and reactive nitrogen species production over time. It is important in the pathophysiology of retinal disorders such as diabetic retinopathy, age-related macular degeneration, retinal detachment, and proliferative vitreoretinopathy, which are the focus of this article. Although the human organism’s defense mechanisms correct autoxidation caused by endogenous or exogenous factors, this may be insufficient, causing an imbalance in favor of excessive ROS production or a weakening of the endogenous antioxidant system, resulting in molecular and cellular damage. Furthermore, modern lifestyles and environmental factors contribute to increased chemical exposure and stress induction, resulting in oxidative stress. In this review, we discuss the current information about oxidative stress and the vitreous proteome with a special focus on vitreoretinal diseases. Additionally, we explore therapies using antioxidants in an attempt to rescue the body from oxidation, restore balance, and maximize healthy body function, as well as new investigational therapies that have shown significant therapeutic potential in preclinical studies and clinical trial outcomes, along with their goals and strategic approaches to combat oxidative stress.

## 1. Introduction

### Oxidative Stress in Retinal Diseases

Oxidative stress (OS) is a common factor in many disorders, including retinal diseases, and is defined as an unbalance between antioxidants and pro-oxidants in favor of the former, leading to a disturbance of redox signaling and/or molecular damage [1]. Despite low amounts of reactive oxygen species (ROS) being necessary for cell homeostasis and redox signaling, increased intracellular concentrations can cause OS [1]. In addition to ROS, other reactive species, such as reactive nitrogen species (RNS) [2], reactive sulfur species [3], reactive electrophile species [4], and reactive halogen species [5], also play an important role in metabolic regulation. The most common ROS and RNS are hydrogen peroxide (H_2_O_2_), superoxide (O_2_^−^), hydroxyl (OH^−^), nitric oxide (NO), and peroxynitrite (ONOO^−^) [1,6]. ROS are produced continuously in mitochondrial oxidative metabolism during cell respiration as a consequence of the use of O_2_ as the final electron acceptor in aerobic organisms [7]. Nevertheless, the excess ROS/RNS production results in an imbalance between oxidants and antioxidants (preventive, sequestering, and repairing) systems, weakens the organism’s defense, and damages essential biomolecules, such as lipids, proteins, and DNA, with negative effects on a variety of organs. Thus, OS can be considered physiological (eustress) when it has specific targets in redox signaling, with a low exposure of organisms. On the other hand, it is considered toxic OS (oxidative distress) when the exposure of non-specific targets to supraphysiological levels of oxidant agents leads to the interruption of redox signaling, with subsequent pathophysiological repercussions [8]. In fact, OS is strongly associated with the onset of different pathologies, including cancer, diabetes, neurodegeneration, and cardiovascular and eye diseases [9]. Retinal diseases, such as diabetic retinopathy (DR), age-related macular degeneration (AMD), retinal detachment (RD), and proliferative vitreoretinopathy (PVR) have been related to higher levels of ROS, affecting a multiplicity of physiological processes, including vascular reactivity and neuronal function. Under various pathological conditions in the retina, ROS-producing systems are activated, which can include enzymes such as NADPH oxidase (NOX), xanthine oxidoreductase, cytochrome P450, mitochondrial cytochrome oxidase, and uncoupled endothelial NOS (eNOS) [10]. The retina is particularly susceptible to OS and lipid peroxidation due to its high metabolic and oxidative phosphorylation rates, high concentration of polyunsaturated fatty acids, and continuous light exposure [11,12]. ROS production imbalance impairs the delicate homeostasis and dynamics of the retinal neurovascular unit since the cells they compose, including vascular cells (pericytes and endothelial cells), the retinal pigment epithelium (RPE), glia, and neuronal cells, are highly susceptible to OS [13]. Coupling these cells as a single and intricate structural network is essential for supplying oxygen and nutrients to the highly metabolically demanding retina, ensuring their normal function and adaptation to varying physiological/pathological conditions [13,14,15]. Under stress conditions such as OS, activated microglial cells and RPE mount an adaptative low-grade inflammatory response, termed para-inflammation, through the release of growth factors and cytokines, and complement activation to restore tissue homeostasis and function. However, in an environment that has sustained oxidative damage, dysregulated para-inflammation gives rise to chronic inflammation mediated by pro-inflammatory cytokines such as tumor necrosis factor-alpha (TNF-α), interleukin-1-beta (IL1β), and interleukin-6, which further increase the production of ROS in RPE cells [16,17,18]. Therefore, although para-inflammation is initially activated in response to OS to maintain rescue photoreceptors, stress-induced chronic inflammatory is the main factor responsible for photoreceptor degeneration [16,19]. Considering the role of OS in the pathophysiology of ocular diseases, the use of compounds with direct or indirect antioxidant activity could represent a beneficial therapeutic strategy to simultaneously reduce inflammation, neurodegeneration, and retinal and vascular dysfunction [18,20,21].

## 2. Oxidative Stress and Vitreous Proteome in Vitreoretinal Diseases

### 2.1. Diabetic Retinopathy

DR is one of the major complications in patients with diabetes mellitus and a leading cause of blindness and visual impairment in middle-aged adults [22]. According to the World Health Organization (WHO), 146 million (34.6%) out of the 422 million adults with diabetes suffer some form of DR [23], but these numbers are expected to grow in parallel with the increased prevalence of DR in middle- and low-income countries [24,25]. The standard treatment for DR is the intravitreal injection of anti-vascular endothelial growth factor (VEGF) agents, or corticosteroids when the response to anti-VEGFs is insufficient [26]. Combination therapy with laser photocoagulation may also be considered. Vitreoretinal surgery is applied for advanced phases of DR [27]. However, these treatments only ameliorate the symptoms.

For many years, DR was considered as a merely microvascular and macrovascular disorder [28,29]. In the early stages (non-proliferative DR), several microvascular changes occur in the eye in response to hyperglycemia, including microaneurysms, thickening of the retinal capillary basement membrane, and subsequent loss of pericytes [30]. Capillary non-perfusion and occlusion gradually drive retinal ischemia, which in turn triggers molecular mechanisms that lead to pathological intraretinal and intravitreal neovascularization in the proliferative phase of DR (PDR) [22,25,30]. Nevertheless, new evidence suggests that neuroglial degeneration may precede microvascular changes as a result of crosstalk between OS, inflammation, glial reactivity, and an imbalance production of neurotrophic factors [29,31]. Due to its multifactorial nature, the pathophysiological mechanisms underlying DR are not fully understood, but it has been suggested that OS plays a central role in metabolic changes triggered in response to chronic hyperglycemia [29,32]. Multiple biochemical pathways have been implicated in ROS production in DR, including the activation of protein kinase C (PKC) and hexosamine pathways, the increased glycolytic flux in the polyol pathway, the production of advanced glycation end products (AGEs), and the upregulation of its receptors (RAGE), as recently reviewed by several authors [32,33,34]. The overproduction of ROS promotes endothelial and mitochondrial dysfunction, inflammatory responses, the activation of microglial cells, and retinal cell apoptosis, which ultimately lead to the appearance of DR clinical features, such as capillary basement membrane thickness, increased vascular permeability, blood–retinal barrier (BRB) breakdown, and neurodegeneration [32,33,35]. On the other hand, it has been shown that photoreceptor cells are a major source of ROS and can contribute to the development of microvascular abnormalities by promoting a pro-inflammatory environment in the diabetic retina [35,36]. Nonetheless, the loss of stressed photoreceptors reversed the OS and inflammation in the retina and attenuated the deterioration of retinal capillaries, thus reducing the severity of DR [35,36]. This supports the role of metabolic stress of photoreceptors on retinal capillary degeneration, which appears to be mediated by the activation and recruitment of nearby cells (e.g., endothelial cells and circulating leukocytes) and release of pro-inflammatory factors [37]. This suggests that the microvascular changes in DR could result from oxidative and metabolic stress in the neural retina [38]. Therefore, the neuroprotection of photoreceptor cells against OS could offer an opportunity for an earlier treatment of DR, even before the appearance of microvascular lesions [35,37]. Despite more clinical trials being required to assess the efficacy of antioxidants as a neuroprotective therapy, they could potentially benefit DR patients (see Section 3) [21,39].

Like other ocular tissues, DR vitreous is affected by metabolic and functional modifications associated with OS. AGEs trigger abnormal crosslinks between collagen fibrils, causing the dissociation from hyaluronan and the destabilization of the gel structure. As a matter of fact, structural and molecular changes in vitreous and at the vitreoretinal surface might exert pathological effects in retinal capillaries, contributing to the progression to PDR [40,41]. Several authors have studied the OS-triggered changes in the vitreous humor proteome in DR/PDR. Higher levels of OS markers such as NO [42,43,44] and AGEs [45,46] were found in PDR vitreous, as well as increased levels of markers of OS damage, including protein carbonylation [45] and lipid peroxidation [47,48]. More recently, Suzuki and co-workers reported the upregulation of a new potential oxidative biomarker, OS-responsive apoptosis-inducing protein, in the vitreous of patients with PDR compared to a non-diabetic group [49]. Furthermore, some authors suggested that a decrease in the antioxidant defenses may be associated with the progression of DR, evidenced by decreased levels of catalase (CAT) [50], superoxide dismutase (SOD) [47], reduced glutathione (GSH) [43], and total antioxidant capacity [51]. On the other hand, Izuta and colleagues detected an increased antioxidant capacity in PDR vitreous and a positive association between it and the levels of VEGF, suggesting that these controversial results may be due to the different types of antioxidant enzymes analyzed in this study [48].

**Figure 1:**
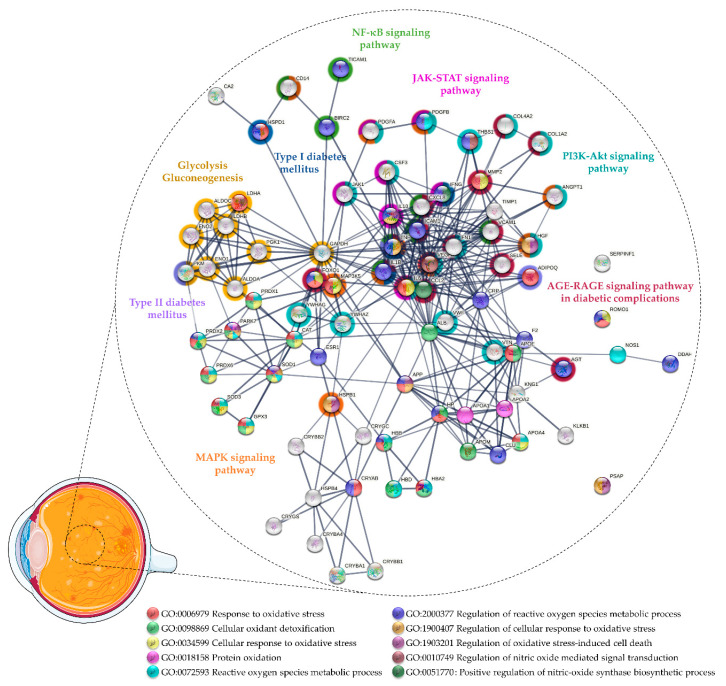
Protein–protein interaction network of the proteins related to oxidative stress found differentially expressed in vitreous collected from patients with DR and proliferative DR (PDR). The network was predicted using STRING 11.5 based on high confidence interaction score and clustered using the Markov Cluster algorithm clustering (inflation parameter: 3).

Proteomics [52,53,54,55,56,57,58,59,60,61,62,63,64] and multiplex ELISA [65,66,67,68,69] studies reported similar outcomes, confirming the impaired antioxidant capacity of vitreous in DR/PDR. Considering the proteins found differentially expressed in these studies, a functional enrichment analysis was performed using STRING v11.5 [70] to gain new insights into the role of OS in DR/PDR and to found potential markers (Figure 1 and Supplementary Table S1). This analysis suggests that the OS increase in DR may be countered by the activation of protective mechanisms through the upregulation of several antioxidant enzymes. Minamoto and co-workers detected CAT only in PDR vitreous, whereas glutathione peroxidase (GPX) was found only in the Macular hole (MH) vitreous [57]. Gao and co-workers reported significantly increased levels of the antioxidant peroxiredoxin-1 (PRDX1) in PDR vitreous and a trend for the upregulation of GPX3 and CAT but, in turn, extracellular SOD (SOD3) was not detected [60]. On the other hand, Zou and co-workers only found superoxide dismutase [Cu-Zn] (SOD1) in PDR vitreous compared with patients with MH [56], which suggests that SOD1 and SOD3 could contribute to different protective mechanisms in DR despite having the same catalytic activity. In addition to SOD1, Zou and co-workers reported the upregulation of other antioxidant proteins (CAT, PRDX) in PDR vitreous after treatment with anti-VEGF drug ranibizumab [56].

Wang and co-workers found lower levels of antioxidants in PDR vitreous, including GPX3 and Parkinson disease protein 7 (PARK7) [62,63]. Loukovaara and co-workers detected higher levels of several OS markers in PDR versus NPDR vitreous, including reactive species modulator 1 and NO synthase (NOS), as well as several antioxidant proteins, such as PRDX2, PRDX6, and CAT [64]. Proteomics analysis also uncovered other antioxidant and neurotrophic proteins in DR/PDR, such as pigment epithelium-derived factor (PEDF), crystallins, or apolipoproteins (Supplementary Table S1 and Figure 1). PEDF is an anti-angiogenic, antioxidant, and neurotropic factor produced by retinal pigment epithelium (RPE) and Müller cells under physiological conditions [71]. Several authors reported unbalanced intravitreal levels of PEDF in DR/PDR; however, the results were inconsistent among studies [52,53,54,58,59,62]. PEDF was initially highlighted for its role as an inhibitor of angiogenesis [72], but its importance as an antioxidant and neurotrophic factor has been demonstrated in several animal models where the lack of this factor leads to an increased susceptibility to retinal degeneration and OS [71,73,74]. It was recently reported that the downregulation of PEDF induced in response to hyperglycemia is countered by the inhibition of AGE-RAGE signaling [75]. In turn, increased levels of PEDF could inhibit retinal hyperpermeability, leukostasis, and angiogenesis by repressing the generation of ROS and the AGE-signaling pathway [76,77,78]. Furthermore, it has been demonstrated that PEDF levels in patients with PDR are associated with the full antioxidant capacity of the vitreous, which suggests that PEDF has a protective role against OS and could be a therapeutic agent for DR/PDR [76].

Crystallins are lens structural molecules belonging to the small heat shock proteins (HSPs) family that also could have a neuroprotective role in DR [79]. Nevertheless, crystallins in DR/PDR could be linked to OS since their expression seems to be induced/repressed at mild and chronic OS conditions, respectively [80]. Accordingly, several members of the crystallin protein family were found to be differentially expressed in PDR vitreous, with some authors reporting their downregulation compared to healthy individuals [62,63], whereas others reported their upregulation in PDR [55,56,81,82]. The link between crystallins expression and OS was further confirmed by data suggesting that αA-crystallin was upregulated in mice after intravitreal injection of a recombinant AGEs protein, suggesting that αA-crystallin may protect photoreceptors against AGE-induced retinal injury [83]. In addition, other studies demonstrated that αB-crystallin could protect retinal cells against degeneration induced by OS [84,85]. More recently, Ghosh and co-workers reported that the loss of βA1-crystallin regulation in glucose metabolism and mitochondrial function of retinal astrocytes induces pathological features similar to DR, such as metabolic abnormalities, OS, and inflammation [82]. Nevertheless, diabetes can induce molecular changes in αA-crystallin, in particular at the phosphorylation level, which can affect its neuroprotective function [86,87]. So, crystallins undoubtedly play a role in DR pathogenesis and offer a potential therapeutic option, although more research will be required to fully understand their mechanism [82,86].

Clusterin (CLU), also known as Apolipoprotein J, is another extracellular chaperone produced by RPE cells that may have a protective role in DR by promoting cell survival and protection from apoptosis [88,89]. CLU showed to protect RPE cells from OS-induced damage, which contributes to cell survival via the PI3K/Akt pathway [90]. In an animal model in diabetes research, CLU expression decreased the levels of pro-inflammatory cytokines, including intercellular adhesion molecule 1 (ICAM1) and vascular cell adhesion protein 1 (VCAM1), and reduced the mitochondrial production of ROS, ameliorating vascular complications associated with diabetes [91]. Nevertheless, CLU has been found to be downregulated in PDR vitreous, suggesting the inhibition of its protective function [56,62,63]. Apolipoprotein E (APOE) could also have a protective role in DR by preventing the aggregation of toxic proteins. Recently, it has been shown that APOE is capable of protecting pericytes against amyloid-induced cytotoxicity linked to the development of type-2 diabetes [92]. Moreover, the lack of APOE has been correlated with an increase in ROS, which reinforces the potential role of OS in DR [93].

In addition to providing potential biomarkers of the vitreous antioxidant state in DR, many proteomics studies highlighted pathways related to OS, evidencing its role in PDR progression and as potential therapeutic targets (Figure 1 and Supplementary Table S1). Glycolysis/gluconeogenesis (KEGG entry (hsa): 00010) is one of the metabolic pathways affected in DR/PDR that seems to be related to OS. As mentioned above, multiple biochemical pathways, which are activated in response to chronic hyperglycemia, have been implicated in ROS production [32,33,34,94]. The glycolytic intermediates accumulated under hyperglycemia/ischemic conditions lead to the dysregulation of glycolysis and oxidative phosphorylation and, eventually, to other neural and microvascular complications observed in DR (reviewed by Yumnamcha and co-workers) [95]. For example, the activation of the polyol pathway increases the uptake of NADPH and NAD+, which is required for GSH regeneration, thus weakening the antioxidant defenses and increasing OS [32,34,94,95]. Meanwhile, the activation of PKC induces the expression of VEGF, endothelin-1, and plasminogen activator inhibitor-1 and the activation of NOX and nuclear factor kappa B (NF-κB) signaling (hsa: 04064), thus contributing to pericyte apoptosis, ROS production, inflammation, and vascular dysfunction [21,32,34,94,95]. AGE-RAGE signaling (hsa: 04933), one of the more enriched pathways according to Kyoto Encyclopedia of Genes and Genomes (KEGG) functional analysis, is another pathway activated in response to chronic hyperglycemia. AGE-RAGE signaling triggers several routes, which ultimately lead to the activation of signaling pathways such as phosphatidylinositol 3-kinase/protein kinase B (PI3K-Akt) (hsa: 04151), Janus kinase-signal transducer and activator of transcription (JAK-STAT) (hsa: 04630), and NF-κB signaling (hsa: 04064) [96]. NF-κB activation induces the expression of a series of pro-inflammatory cytokines [97], many of which were found to be upregulated in PDR vitreous, as described by Schori and colleagues [55]. Likewise, other authors reported the upregulation of TNF-α) [68], TIR domain-containing adapter molecule 1 (TICAM1) [64], baculoviral IAP repeat-containing protein 2 [64], IL1β [68,69], interleukin 8 [65,66,68], monocyte differentiation antigen CD14, and adhesion molecules (ICAM1, VCAM1) [55,66,67]. Many of these pro-inflammatory cytokines (TICAM1, IL1β, ICAM1), among other proteins (e.g., angiotensinogen, AGT), were related to the regulation of NO biosynthetic process (gene ontology (GO) entry: 0045428) in a functional analysis. The NO biosynthetic process is enhanced in response to the activation of NF-κB, which eventually leads to the apoptosis of endothelial cells exposed to hyperglycemia-induced OS [98]. The biosynthesis of NO is catalyzed by different isoforms of NOS, including constitutively expressed neuronal NOS (nNOS) and eNOS, and also inducible NOS (iNOS) [99], which is triggered under inflammatory and pathological conditions [99,100,101]. Under OS, cytokines such as IL1β, TNF-α, and interferon-gamma induce the synthesis of iNOS, a process that was shown to be reversed by antioxidants (e.g., CAT) [102]. Nevertheless, in proteomics studies, only nNOS, the main source of NO from retinal neurons in early DR, was found to be upregulated in PDR vitreous [64]. On the other hand, it has been suggested that eNOS, mainly expressed in the vascular endothelium, could trigger neovascularization by inducing VEGF and PGE2 [103]. Despite the increased levels of eNOS found in active diabetic fibrovascular epiretinal membranes (ERM) [104], it has been detected neither in the PDR vitreous nor in non-diabetic controls [103]. In any case, impaired eNOS expression has been linked to DR features such as changes in vascular permeability and the breakdown of the BRB [105,106]. Additionally, several pieces of evidence reinforce the idea that increased activity and levels of iNOS drive the increased BRB permeability, glial reactivity, and retinal degeneration observed in DR [44,107,108,109,110]. Enhanced NO production by iNOS has been implicated in various pathological processes, including inflammation, tissue damage, and cell apoptosis in an inflammatory and/or ischemic environment [101]. In addition, it was reported that the inhibition of iNOS by blocking angiotensin II (Ang II) type 1 receptor (AT1R) ameliorated glial activation and OS in animal models in diabetes [109]. These findings suggest a link between the NO and renin–angiotensin system (RAS), which are implicated in the progression of DR, and provide a potential therapeutic target against OS [100].

To counteract the negative effects of the activation of these pathways in response to OS, protective mechanisms seem to be triggered to modulate key pathological processes in DR. Nuclear factor erythroid 2-related factor 2 (Nrf2) is a redox-sensitive transcription factor that regulates the expression of genes encoding many antioxidant enzymes and phase II detoxifying enzymes, representing one of the major cellular defenses against OS [111]. It has been reported that diabetes increases retinal Nrf2 but decreases Nrf2 DNA-binding activity [112]. Gardner and Sundstrom [113] showed evidence that the Nrf2-mediated OS response is significantly activated in PDR, which is reinforced by the increased intravitreal expression of SOD2. However, other antioxidant proteins such as CAT, PRDX, and SOD1 and SOD3 were found to be downregulated in PDR vitreous compared to MH/ERM [113]. The potential therapeutic effect of Nrf2 was also supported by Deliyanti and co-workers, who showed that its stimulation prevents the increase in vascular permeability and the upregulation of angiogenic and inflammatory mediators induced by hyperglycemia [114].

### 2.2. Age-Related Macular Degeneration

AMD is a multifactorial ocular disease that affects the central retina and represents the leading cause of blindness in developed countries [115,116]. According to the WHO, the global burden of AMD is predicted to increase, foreseeing that 288 million people will be affected by the disease by 2040 [23,115]. AMD is categorized into two distinct forms, a “dry” or non-exudative form, and a “wet” or neovascular AMD (nAMD) [117]. In the early phase, yellowish deposits (drusen) are accumulated underneath the retina, accompanied by the infiltration of microglia and choroidal macrophages, thickening of Bruch’s membrane, and changes in RPE pigmentation [116,118]. The non-exudative form develops at more advanced stages of dry AMD and is characterized by overlying regions of RPE and photoreceptor cell degeneration [117,118]. The nAMD form accounts for only 10–15% of all cases but it is responsible of the most severe cases. At this stage, the patients present choroidal neovascularization (CNV) associated with vascular leakage and the breakthrough of these brittle vessels, which eventually leads to outer BRB breakdown, retinal and vitreous hemorrhage, fibrosis, and photoreceptor loss [116,118]. Currently, the treatment of these patients at this phase is based on anti-angiogenic drugs, but no therapy is yet available for “dry” AMD [119,120].

Even though AMD pathogenesis is not completely understood, OS has been identified as a key factor in the onset of this disease. As matter of fact, factors that contribute to OS, such as polymorphisms in antioxidant enzyme genes, cigarette smoke, and exposure to sunlight, are considered risk factors for AMD [120,121]. In recent years, multiple mechanisms have been evaluated to explain the role of OS in AMD [122,123,124]. The combination of a high demand for energy and oxygen, intensive light exposure, the rich content in polyunsaturated fatty acids (PUFAs) of the outer segment of photoreceptors, and the presence of photosensitizers makes the retina very susceptible to lipid peroxidation [17,125]. RPE is particularly important for maintaining retinal homeostasis and protecting from OS since it captures the excess light and removes the oxidized PUFAs and other waste products through different mechanisms [126,127,128]. Under acute OS, the autophagic activity becomes augmented in RPE cells as a protection mechanism. Nevertheless, chronic OS can in turn contribute to dysfunctional autophagy by blocking the function of lysosomal enzymes and the hyperpermeabilization of lysosomal membranes [128,129,130]. Impaired autophagy contributes to the formation of drusen [131,132], preventing the influx of oxygen and nutrients to the photoreceptors and the removal of the waste between RPE and choroid [133]. This disturbs the metabolic co-dependence ecosystem between the RPE and the photoreceptors, which ensures their proper function, suggesting that metabolic reprogramming could underlie retinal degeneration in AMD [134,135]. Several reports indicate that the overactivation of the mammalian target of rapamycin (mTOR) pathway may be the link between OS response, autophagic dysfunction, and metabolic reprogramming in AMD [129]. Increased activation of mTOR has been reported in RPE from AMD donors when compared with RPE cells from non-AMD eyes [136]. Although the mTOR pathway is activated in response to stress and starvation to promote RPE cells survival, its overactivation inhibits autophagy, induces mitochondria damages, and RPE dedifferentiation [129,136]. In turn, the chronic activation of mTOR prevents the return to baseline of glycolytic metabolism in RPE, leading to the nutrient deprivation of photoreceptors and further neurodegeneration [137,138]. Therefore, targeting the pathways associated with OS by reinforcing the supply of antioxidants and energy to photoreceptors could tip the balance toward the activation of antioxidant defense mechanisms in AMD [123]. Although therapies for “dry” AMD are still an unmet requirement, the consumption of antioxidant supplements in the context of a healthy diet has been recommended for the prevention of risk factors [139,140,141].

**Figure 2:**
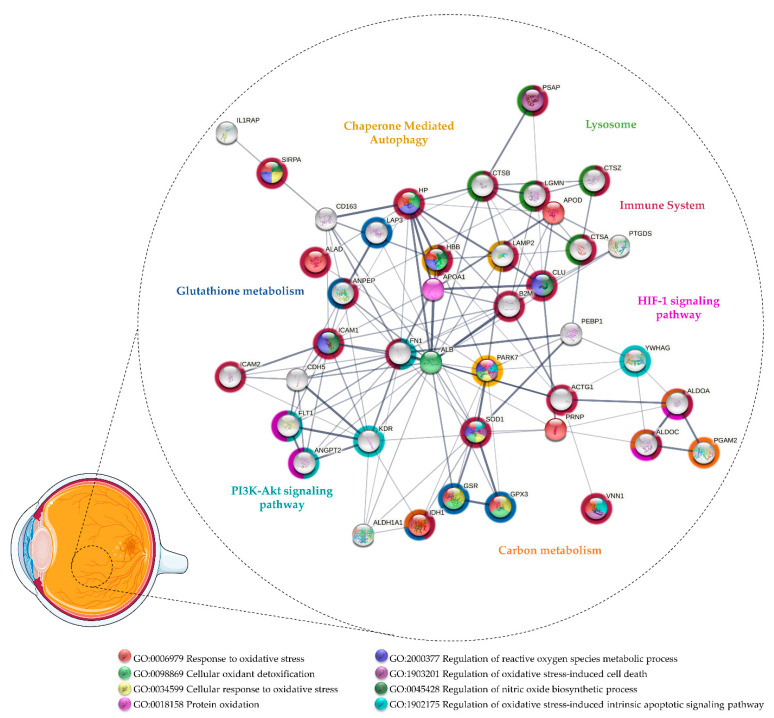
Protein–protein interaction network of the proteins related to oxidative stress found differentially expressed in vitreous collected from patients with age-related macular degeneration. The network was predicted using STRING 11.5 based on medium confidence interaction.

Although few studies have focused on vitreous proteomics in AMD, some potential biomarkers were provided [55,142,143]. Thus, the proteins found differentially expressed in these studies were considered for functional enrichment by STRING v11.5 [70] to identify potential biomarkers of OS and functional partnerships (Figure 2 and Supplementary Table S2). Specific pathways were found to be enriched, suggesting that compensatory mechanisms are triggered in response to OS. This fact is more evident at advanced stages of AMD, since several proteins associated with cellular oxidant detoxification were found to be upregulated in nAMD. Koss and co-workers reported higher levels of GPX3 and haptoglobin (HP) in the nAMD vitreous compared to patients with idiopathic floaters, proposing that their upregulation is due to the activation of detoxification mechanisms in response to OS [142]. GPX3 protects the retina from oxidative damage through the GSH metabolism (hsa:00480), which has been found to be affected in AMD according to a functional analysis based on the KEGG database. As recently reviewed, mitochondrial GSH removal in RPE and retina has been related to mitochondrial dysfunction and increased RPE cell death, emphasizing that the supply of GSH and the reinforcement of the glutathione antioxidant system could provide a potential therapeutic strategy for maintaining retinal homeostasis in AMD [144]. HP is an acute-phase protein that plays a relevant detoxification role by capturing free hemoglobin, protecting tissues against OS-induced damages [145]. It has been suggested that HP is expressed in the retina as an early protective mechanism against iron-mediated ROS formation, regulated by cytokines, such as IL1 and IL6 [146]. Therefore, HP could be also involved in the mediation of inflammatory processes in AMD, which is suggested by a functional analysis considering their role in immune system signaling (Reactome entry: R-HSA:168256). HP levels were found to be significantly increased in the macula with AMD when compared to controls [147], but its increase was non-significant in the serum of AMD patients [148]. Furthermore, although the HP phenotype 1–1, recognized for its better binding efficiency to hemoglobin and antioxidant action, has reported to be protective against DR, the same has not been verified for nAMD [149].

Nobl and colleagues identified four potential biomarkers by comparing nAMD vitreous with different degrees of CNV [143]. Of these, they validated the upregulation of CLU and PEDF in nAMD [143], proteins that can play a protective role against OS and, hence, could be therapeutic agents in AMD such as in DR/PDR (see Section 3.1). Other authors reported decreased intravitreal levels of PEDF associated with CNV in AMD patients [150,151]. CLU has a cytoprotective effect, reducing apoptosis and OS in the retina [88,89,90]. Beyond vitreous, CLU was found to be upregulated in the aqueous humor [152], aqueous humor exosomes [153], macular Bruch membrane/Choroid [154], and drusen [155] of AMD donors. CLU has also been detected in the drusen from healthy/older donors [156,157], but it has been suggested that this accumulation is a response to injury or stress of the RPE or choriocapillaris [88,155]. As matter of fact, it was reported that RPE cells from AMD donors secrete more CLU compared to healthy control donors [158]. Although their role in ocular diseases is not well understood, multiple functions have been attributed to CLU [88,89]. This apolipoprotein is capable of inhibiting the formation of toxic beta-amyloid (Aβ) fibrils [159,160,161], known to increase the secretion of cytokines, triggering a pro-inflammatory environment in the retina and destabilization of RPE tight junctions [162,163,164,165]. More recently, it has been proposed that the autophagy activation in RPE cells may be a protective mechanism to reduce the accumulation of aggregated toxic proteins in the retina, such as Aβ [165]. This process can be assisted by chaperones, such as CLU, through chaperone-mediated autophagy (CMA; R-HSA:9613829) [132,166], which is one of the biological processes inferred in the functional analysis. Furthermore, both Aβ and CLU [88] can further contribute to AMD pathogenesis through their potential role in the regulation of the complement system. While Aβ peptides have been implicated in the activation of complement cascades [162,167,168], CLU is a negative regulator of the cytolysis mediated by complement activation [88,89]. Indeed, several complement components were detected in drusen and some genetic variants (e.g., Complement C3, factor H, and factor B) are considered to be risk factors for AMD [169,170,171,172,173]. Complement activation contributes to the recruitment and activation of immune cells, expression, and secretion of several pro-inflammatory cytokines and growth factors (e.g., VEGF), increased OS, and the accumulation of lipids in the retina [172,173,174]. On the other hand, it was hypothesized that CLU secretion may contribute to Aβ production by RPE cells and drusen formation [175]. Nevertheless, CLU seems to play a relevant role in AMD pathogenesis, but further studies are required to understand if it is a protective or pathogenic factor.

Interestingly, Schori and colleagues compared the vitreous from dry AMD and nAMD patients with ERM controls using a label-free LC-MS/MS quantitative method [55]. They found that several antioxidant proteins were significantly upregulated in nAMD, such as SOD1, glutathione reductase (GSR), and PARK7. However, SOD1 and other antioxidant enzymes such as GPX3, SOD3, and CAT only showed a trend for upregulation in dry AMD, which suggests that protective mechanisms (e.g., Nrf2 pathway) are activated with the progression of the disease [55]. As seen in Supplementary Table S2 and Figure 2, SOD1 and PARK7 were associated in the functional analysis as positive regulators of the OS-induced intrinsic apoptotic signaling pathways (GO: 1902175; GO: 1902177). As previously mentioned, PARK7 protects photoreceptors and RPE cells against OS by transcription regulation and ROS scavenging. The lack of PARK7 has also been linked to retinal/RPE degeneration in response to OS [176,177,178]. SOD1-deficient mice also exhibit pathological features found in AMD, including drusen, thickened Bruch’s membrane, and CNV [179]. Therefore, the loss of gene function of these antioxidant proteins provides a link between OS and neurodegeneration in the retina [180]. It was demonstrated recently by Zhu and co-workers that SOD1/PARK7/Parkin triple knockout exhibits retinal degeneration with aging [181]. Moreover, Nrf2-deficient mice also developed pathological hallmarks similar to human AMD, including deregulated autophagy, the accumulation of drusen, and RPE degeneration [182]. Considering that PARK7 and SOD1 have been correlated with the Nrf2 pathway [180], their upregulation in nAMD implies the activation of this defense system against OS [180,183,184]. In addition to proteins related to the Nrf2 pathway, Schori and colleagues [55] reported differentially expressed proteins implicated in processes associated with OS, including CMA (R-HSA: 9613829), GSH metabolism (hsa: 00480), HIF-1 signaling (hsa:04066), and PI3K-Akt signaling (hsa: 04151). Lysosome-associated membrane glycoprotein 2 (LAMP2), upregulated in nAMD, is a lysosome marker that may play a protective role in AMD as it mediates autophagy/CMA clearance through the regulation of autophagosomes and lysosomes (autolysosome) [185]. Under OS, an increase in the autophagic activity is required to assist the digestion and removal of damaged and oxidized material within autolysosomes, performed by lysosomal acid hydrolases such as cathepsins [166], which are also upregulated in nAMD [55]. In addition, the loss of LAMP2 function compromises phagocytic and lysosomal degradation, leading to the increased exocytosis of defective materials in RPE cells and the accumulation of basal laminar deposits [185]. Regarding the proteins involved in glutathione metabolism, GSR has been found to be upregulated in nAMD, but isocitrate dehydrogenase [NADP] cytoplasmic (IDH1) and aminopeptidases were found to be downregulated in dry AMD. IDH1 is essential for efficient glutathione recycling since it provides NADPH to form GSH by GSR [186]. Indeed, the activation of the Nrf2 pathway regulates the NADPH-producing enzymes, such as IDH1, thus modulating its levels [187,188]. An IDH1 increase in nAMD compared to ERM controls and dry AMD was detected, but this difference was not statistically significant. Considering the contribution of OS in AMD, it is likely that the Nrf2 represents a chief regulator of antioxidant protective mechanisms [55]. Thus, as in PDR, the activation of the Nrf2 pathway could be a suitable therapeutic approach, particularly in dry AMD, for which no therapy is available.

### 2.3. Retinal Detachment and Proliferative Vitreoretinopathy

RD is a potentially blinding disease characterized by a physical separation between the neurosensory retina and the RPE [189]. Rhegmatogenous retinal detachment (RRD) represents the most common type of RD [189], with an incidence of 13 cases per 100,000 people per year [190,191]. In developed countries, 3.9% to 13.7% of all patients with RD develop PVR, a complication that represents the most common cause of failure in RRD surgery [192,193]. PVR results of exacerbated vitreoretinal wound-healing in response to trauma, RD, and surgical interventions at the vitreoretinal interface, leading to the formation of fibrovascular membranes and vitreoretinal traction [193,194]. Currently, the management of these diseases is exclusively surgical, with primary success rates of up to 90% [195,196]. Nevertheless, when RRD is associated with PVR, patients experience worse visual recovery and reattachment rates [197]. Numerous drugs have been proposed for the prevention of PVR or recurrence after surgery, including corticosteroids, anti-neoplastic/anti-proliferative, anti-VEGF, and antioxidant agents but, until now, no proven pharmacologic agents have been incorporated routinely into clinical practice [193,198].

In most cases, RRD arises as a consequence of the liquefaction and degeneration of the vitreous, which is initiated by several factors (e.g., aging or trauma) that induce the disintegration of collagen structural network and reduce vitreoretinal adhesion, leading to the accumulation of vitreous fluid in the subretinal space [199,200]. OS is one of these factors, suggesting that an imbalance between the levels of ROS and antioxidants can be underlying vitreous liquefaction and RRD [201]. Some authors reported higher levels of OS biomarkers [202], but lower antioxidant capacity [203], in the vitreous of patients with RRD when compared to MH patients. Higher levels of OS markers in the vitreous have been related to RRD severity [202], whereas the extent of the detached area in RRD was positively correlated with biological antioxidant potential in the vitreous fluid [203]. Although no significant differences in the antioxidative capacity of the vitreous between patients with RRD and ERM/MH were found, the increase in the total antioxidant status of vitreous is correlated with the duration of RRD and probably with the progression to PVR [204]. Beyond this, the physical separation of the neurosensory retina and the underlying RPE interferes with the supply of oxygen and nutrients, thus leading to an ischemic environment, lack of neurotrophic factors and, eventually, the death of photoreceptors and neurons. Under hypoxic and metabolic stress, photoreceptors undergo mitochondrial dysfunction, which contributes to the overproduction of ROS and a higher susceptibility to OS [194,205]. Furthermore, the BRB breakdown leads to an influx of growth factors and inflammatory mediators, which increases the chemotactic and mitogenic activity in vitreous and foments leukocyte infiltration into the injury site [192,194,206]. The oxidative burst of neutrophils and macrophages further increases the release of ROS, neurotoxic enzymes, and cytokines [193]. In turn, cytokines stimulate the activation of macrophages, Muller cells, and microglia, which also contribute to the cytotoxic effect mediated by OS on the photoreceptors after RD [194,207]. The exposition to cytokines and growth factors, changes in cell adhesion, and the loss of signaling resultant from retinal cells death provoke the epithelial–mesenchymal transition (EMT), an essential pathological process in the development of PVR [193,208] that results in ECM remodeling, preventing the reattachment of the retina [209,210]. As in other pathological processes, OS plays a role in EMT, which contributes to the pathogenesis of PVR [211]. A synergistic effect between transforming growth factor, macrophage migration inhibitory factor, and H_2_O_2_ induces EMT through the upregulation of α-smooth muscle actin, vimentin, and fibronectin and the downregulation of cadherins [211,212]. So, considering the central role of OS in many of these pathological mechanisms, it has been suggested that antioxidants may attenuate photoreceptor death after RD and delay its progression to PVR [213,214,215].

**Figure 3:**
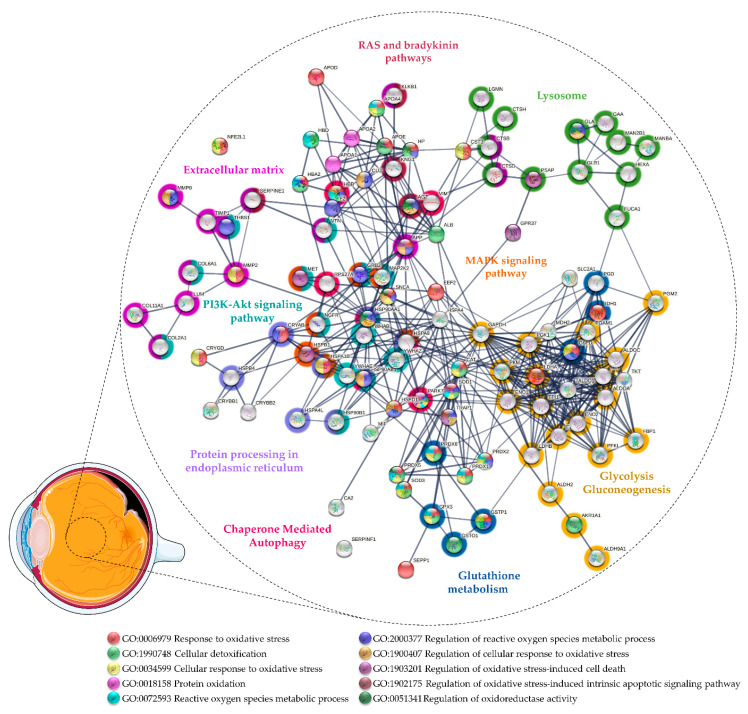
Protein–protein interaction network of the proteins related to oxidative stress found differentially expressed in vitreous from patients with rhegmatogenous retinal detachment (RRD) and proliferative vitreoretinopathy (PVR). The network was predicted using STRING 11.5 based on high confidence interaction score and clustered using the Markov Cluster algorithm clustering (inflation parameter: 3).

The first studies on vitreous proteome were focused on PVR [59,216,217,218] and only recently more attention has been directed to the pathological mechanisms underlying RRD pathogenesis [219,220,221,222]. These studies shed some light on the role of OS in these pathologies (see Supplementary Table S3 and Figure 3). Yu and co-workers conducted some of the first proteomics approaches in the study of human vitreous samples collected from RRD patients with PVR [216,217,218]. Detoxification proteins, such as SOD1, PARK7, PRDX1, and PRDX2, were only detected in vitreous from healthy donors, whereas SOD3, GPX3, and HP were only detected at more severe PVR grades [216]. This suggests that a reduction in the antioxidant capacity in the eye could be involved in the onset of PVR, but protective mechanisms against OS can be triggered in more advanced states. For example, SOD1 was only detected in healthy donors, while SOD3 was only detected in severe PVR, which reinforces, like in PDR (see Section 3.1), that these proteins play different roles despite having the same catalytic activity. Yu and co-workers also found differential levels of Aβ, AGT, and neutrophil collagenase (MMP8), proteins associated with the regulation of ROS biosynthetic process (GO: 1903426), including the NO biosynthetic process [216]. High intravitreal levels of NO and NO pathway metabolites have been implicated in RD/PVR [43,223] and associated with various pathological processes (see Section 3.1). Therefore, the inhibition of NOS and scavenging of ROS could provide a neuroprotection mechanism against OS by potentiating the effect of neurotrophic factors [224]. Aβ was only detected in moderate PVR in this study, while AGT and MMP8 were found in severe PVR [216]. It has been shown that Aβ is expressed in response to elevated ROS [225]. In turn, Aβ stimulates NO biosynthesis through the induction of iNOS, providing a correlation between OS and Aβ-induced damages, such as mitochondrial dysfunction, neurotoxicity, inflammation, and neuronal apoptosis [225,226,227].

MMP8 is constitutively expressed and stored in the secretory granules of neutrophils and eosinophils [228,229]. MMP8 is mainly present in its latent form (pro-MMP8) [229], but NO and its reactive intermediates are capable of converting MMP8 into its active form, which could be inhibited by antioxidant proteins such as SOD or CAT [230]. Once activated, MMP8 cleaves a wide range of substrates, including fibrillar collagens (types I, II, III), proteoglycans, serine protease inhibitors, and several chemokines [228,229]. An imbalance in the levels of matrix metalloproteins and their inhibitors (e.g., TIMP) within the detached retina and vitreous has been suggested to have a role in RRD and PVR membrane formation [231,232]. Indeed, the levels of MMP8 in vitreous/subretinal fluid have been correlated with pathological features, such as the duration and extent of RD [233,234] and the grade of postoperative PVR [235]. AGT is an important component of the RAS, which is responsible for the control of retinal vasculature (recently reviewed by Phipps [236] and Holappa [237]) and, therefore, its dysfunction has been correlated with some vitreoretinal diseases. AGT is cleaved into several effector peptides through different cleavage pathways, but the best known is the conversion to Ang II via the renin/angiotensin-converting enzyme (ACE). Ang II has a vasoconstrictor effect, through cell proliferation and fibrosis, or a vasodilator effect, depending on whether the activation is via AT1R or receptor 2 (AT2R), respectively. Even thus, its role in the regulation of retinal vasculature and the maintenance of the neurovascular unit is not fully understood [236,237]. Nevertheless, Hoerster and co-workers hypothesized that the inhibition of ACE could counteract EMT and, eventually, fibrosis in PVR. However, the administration of ACE inhibitors did not show any effect on the levels of pro-fibrotic cytokines/growth factors [238]. Notwithstanding this, ACE inhibitors can also affect the bradykinin breakdown in the kallikrein–kinin system [239,240] which has also been implicated in PVR by Yu and co-workers [216,217,218]. They found higher levels of kininogen 1 in severe PVR when compared to moderate cases, suggesting a potential biomarker of the disease severity [216,217,218]. Kininogen is converted by kallikrein into kinin-free kininogen, which has anti-proliferative and anti-angiogenic properties, and bradykinin, which promotes vasodilation through the release of tissue plasminogen activator, NO, and prostacyclin from endothelial cells [239,240]. Thus, it may have beneficial effects by preventing PVR events, such as ECM deposition, cell proliferation, and by protecting the retina against ischemic damage [241,242,243]. On the other hand, bradykinin could further contribute to the pathology by inducing the expression of inflammatory mediators and NO formation and increasing retinal vascular permeability, which could lead to retinal edema and intraocular hemorrhage [241,243]. So, further studies are needed to understand the role of the RAS and kallikrein–kinin systems in PVR.

Regarding vitreous studies on RRD, Wu and co-workers found 103 proteins differentially expressed by comparing samples from patients with RRD, associated with choroidal detachment, using iTRAQ labeling. Of these, proteins associated with response to OS (GO:0006979) and cellular detoxification (GO: 1990748) were found to be upregulated, including HP and apolipoproteins A4 and D. On the other hand, proteins with chaperone activity such as APOE and CLU were downregulated. These proteins may have a protective role against OS-induced damage and amyloid-induced cytotoxicity in retinal diseases (see Section 3.1). Of note, Aβ was found to be downregulated in this study. Higher levels of kininogen 1 and plasma kallikrein were found in RRD associated with choroidal detachment than in RRD, reinforcing the potential role of kallikrein–kinin systems in PVR pathogenesis [244]. Our research group compared the vitreous proteome in RRD with ERM using iTRAQ labeling [219]. Of the 150 proteins reported as differentially expressed, several detoxification enzymes were found to be upregulated in RRD, including PRDX1, PRDX2, glutathione S-transferase (GSTP1), and aldo-keto reductase family 1 member A1 (AKR1A1). GSTP1 is an intracellular detoxification enzyme expressed in the retina, iris, and cornea that plays an important role in glutathione metabolism (see Section 3.2) [144,245,246,247]. Its overexpression in RPE cells protects them against H_2_O_2_-induced mitochondrial damages and death [248]. We also found higher levels of several proteins of the crystallin family, including α- and β-crystallins, which could have a role in the response to OS. We hypothesized that their overexpression could represent the activation of a protective mechanism against retinal cell death in RD [219], as mentioned above in Section 3.1 [84,85]. Recently, Hamadmad and co-workers confirmed our results, both at the transcriptional and translational levels, showing that αA-crystallin is upregulated after RD and that its increase is time dependent [249]. More recently, Öhman and co-workers compared the vitreous proteome of RRD patients with other vitreoretinal diseases, including MH, ERM, and PDR using SWATH-mass spectrometry [220]. They identified and quantified a total of 1177 proteins across all 151 vitreous samples, which represents, to our knowledge, the largest dataset of the RRD vitreous proteome. They confirmed our previous results, finding higher levels of AKR1A1, PRDX1, PRDX5, PRDX6, and glutathione metabolism-associated enzymes (GSTP1, GSTO1) in RRD when compared to other diseases. Additionally, they reported the upregulation of other detoxification enzymes including CAT, SOD1, and PARK7. This study also confirmed our reported upregulation of HSPs, which can represent a mechanism to protect retinal cells against ischemia and OS. The upregulation of HSPs can prevent apoptosis, promoting cell survival by several mechanisms, including the activation of the PI3K-Akt signaling pathway (hsa: 04151) or the increase in cell antioxidant potential (e.g., higher GSH levels) [250,251,252]. The upregulation of HSPs and other proteins associated with CMA (R-HSA: 9613829) and the ubiquitin–proteasome system (WikiPathways entry: WP2359) in both studies [219,220] may suggest the triggering of autophagy during RRD. The downregulation of autophagy has been associated with a reduction in photoreceptor survival after RD [253]. Recently, Xiao and co-workers showed that the inhibition of autophagy in rod photoreceptors increased their apoptosis and necroptosis during RD and diminished the expression of key aerobic glycolysis intermediates. This suggests that functional autophagy maintains photoreceptors’ homeostasis, glycolysis, and survival under oxygen and nutrient deprivation [254]. Interestingly, several glycolytic enzymes were reported as upregulated in RRD vitreous both by our research group and Öhman and co-workers [219,220], which implies that retinal cells increase their metabolism after RD. On the other hand, Yu and co-workers reported lower levels of several glycolytic enzymes in moderate and severe PVR [216]. Therefore, this suggests that the glycolytic process is activated after RD to compensate for the metabolic stress of retinal cells, but its reduction may lead to the death of photoreceptors at more severe stages of PVR. In conclusion, the regulation of the glycolytic process may represent a potential therapeutic strategy to limit photoreceptor death after DR.

## 3. Targeting Oxidative Stress/the Treatment by Inhibition of Oxidative Stress

Several pathways are implicated in ROS production and increased OS in retinal diseases. RAS is a significant route pathway in retinal diseases, and drugs targeting Ang II are under research. High levels of prorenin and Ang II have been identified in the vitreous of diabetic patients [255]. Ang II contributes to diabetic retinal complications by activating the AT1R/AT2R in blood vessels and retinal neurons and may trigger NOX via AT1R, causing ROS production in retinal blood vessels and Müller cells, resulting in retinal vascular dysfunction and neurodegeneration [256]. Other causes of an increase in ROS include structural changes in macromolecules (DNA, proteins, and lipids), which generate permanent chemical reactions that cause oxidative damage to the human body. However, unexpected electron release and interaction with molecular oxygen are major contributors to ROS generation [257]. Other ROS are produced in excess by the action of exogenous factors representing stress to the human organism, such as environmental pollution (air, soil, and water), radiation of all types (UV, gamma, etc.), toxic habits (tobacco, alcohol, and drugs), poor diet, the exposure to toxic substances (fertilizers and pesticides), metabolism of some medicines and high physical or psychological stress [258]. Although controversial, there is another way of ROS production as a consequence of genetic alterations (hereditary or not) or physiological disorders of the organism. However, some authors consider the excessive production of ROS, either endogenous or exogenous, as a consequence of genetic alterations or physiological disorders [259]. Although antioxidant mechanisms are available to reduce the excess of ROS, when these defenses are insufficient an imbalance occurs in favor of excessive ROS production or the weakening of endogenous antioxidant systems [260]. To summarize, reducing OS can be achieved by decreasing exposure to environmental pollutants with oxidative properties, by increasing levels of endogenous and exogenous antioxidants, or by decreasing the generation of ROS. In addition to the elimination of toxic habits, such as alcohol and tobacco consumption, and the increased consumption of vegetables and fruit, the introduction of supplements with antioxidant properties in the diet has increased lately with the aim of counteracting the harmful effects of OS. In fact, high antioxidant levels may eliminate or prevent the formation of ROS However, studies on the effect of antioxidant diet supplements are inconclusive or inconsistent, suggesting that they do not offer sufficient protection against OS. We are left with the third option of suppressing oxidative damage, which appears to be the key to success in the fight against OS.

### 3.1. Antioxidants

An antioxidant is a compound that can reduce or inhibit the oxidation of other molecules. Oxidation is a chemical reaction in which electrons or hydrogen are transferred to an oxidizing agent. Free radicals produced during oxidation reactions initiate chain reactions that, when occurring inside the cells, might harm or kill them. Antioxidants remove the free radicals and inhibit additional oxidation processes [261]. The optimal antioxidant treatment would be one preventing the production of excess ROS, stimulating the reparation processes of tissues damaged by ROS attack, and increasing the antioxidant capacity of the human organism by supplying components capable of removing excess ROS. Eating fresh fruits and vegetables regularly is one of the strategies to prevent excessive ROS production and should be the first step in the treatment approach; nevertheless, it is not always a sufficient response to excessive ROS production [262]. The topic of antioxidant therapy is still a matter of debate, being the discussion focused on the administration of food supplements containing antioxidant compounds. Experimental evidence on the relationship between OS and disease progression is overwhelming, particularly in chronic disease. However the therapeutic administration of antioxidant products is frequently regarded as supplemental or secondary [260]. Current regulations consider antioxidants as nutritional supplements or natural health products rather than medications because OS is not recognized as a clinical category [263]. Studies show that dietary supplementation with foods rich in carotenoids (especially lutein and zeaxanthin) can increase macular pigment concentration and density. Their protective effects include absorption of harmful wavelengths that are associated with photochemical damage to the sensory retina as well as the removal of free radicals and ROS generated by metabolic activity [264]. However, studies on the anatomical and functional benefits appear to be controversial. A five-year follow-up study (The Beaver Dam Eye Study), showed a modest link between zinc intake and decreased risk of early AMD [265]. In a similar study (Blue Mountains Eye Study), there a significant association between antioxidant intake and the incidence of early AMD was not found [266]. Noteworthy, antioxidant doses in this study were lower than in a third study (Age-Related Eye Disease Study, AREDS) [267]. AREDS demonstrated that a combination of antioxidants, such as vitamin C 500 mg, Vitamin E 400IU, Beta-carotene 15 mg (or Vitamin A 25,000 IU) plus zinc oxide 80 mg, and cupric oxide 2 mg, decreased the risk of progression to the advanced form of AMD by 25% and reduced the risk of decreased vision by 27% [267].

It is worthwhile to acknowledge that the arsenal of antioxidant therapy does not only include antioxidant substances, but also non-classical agents, such as metal chelators (for example, serum EDTA, D-spherrosamine, among others), oxidant agents whose mechanism is based on the stimulation of endogenous antioxidants. Table 1 provides a brief overview of antioxidants, molecules essential for the action of various antioxidant enzymes, and enzymatic systems for reducing OS.

### 3.2. Investigational Therapies

Several antioxidant therapy approaches are currently being researched, some of which are in the clinical trial stage. These new therapies aim to prevent the formation of ROSor/and promote its removal. The elimination of O_2_^−^ prevents the formation of ONOO^−^, while the remotion of H_2_O_2_ prevents the formation of OH^−^ and HOX. Other investigational therapies consist of the administration of enzymes (e.g., SOD) that remove H_2_O_2_ and lipid hydroperoxides, preventing the formation of other ROS. Several different strategies are being investigated to combat OS, such as the increase in GSH using its precursors, the stimulation of the synthesis of antioxidant enzymes through the activation of Nrf2, the inhibition of NOX, and the supplementation of dietary antioxidants, among others [284].

Clinical trials have shown that preventing ROS formation can reduce the retinal damage associated with the vascular breakdown and neurodegeneration observed in DR and AMD [285]. Numerous molecules have been evaluated as antioxidants in preclinical studies, as summarized in Table 2, suggesting that there is an important therapeutic potential. The effectiveness of activators of Nrf2, such as PB125, in decreasing OS in age-related diseases has been studied in clinical assays [286]. As previously mentioned, the activation of Nrf2 has great potential as a therapeutic approach for the treatment of retinal diseases, particularly neurodegenerative diseases [111]. In addition to protecting the retina against the OS, Nrf2 activators demonstrated great efficiency in reducing inflammation, vascular permeability, and neuronal degeneration in animal/cells models of glaucoma [287,288], DR [114,289], and AMD [290]. However, the results of clinical trials with antioxidants are inconclusive to date. One of the main reasons for this may be the use of a single agent in clinical trials with antioxidants, challenging the ability to therapeutically apply antioxidant strategies. One limitation of the single use of antioxidants depends on whether OS plays a primary or secondary role in a specific disease. If secondary, the antioxidant agent may not significantly impact disease progression, and therefore, the extent to which these agents may be used to ameliorate some symptoms should be assessed [263]. Another important limitation is the fact that certain molecules do not work per “si” but in a chain of reactions, leading to them potentially not being so efficacious in vivo [263]. Lastly, as retinal diseases are typically multifactorial diseases, patients could benefit from the combined administration of several exogenous antioxidant compounds or antioxidants with other drugs [291]. However, since these formulations could have synergistic therapeutic effects, it is difficult to isolate the effect of a specific antioxidant molecule by itself or generate certain antioxidant therapy combinations, which can make it difficult to adjust the recommended dose for each patient. Therefore, the safety and effectiveness of these formulations and/or purified natural antioxidants should be assessed in clinical trials or pharmacovigilance studies [291]. All these limitations make conducting a clinical trial very challenging.

## 4. Conclusions

Research on oxidative stress has been of increasing importance for understanding the development of vitreoretinal diseases. This review summarizes the results of proteomics studies with the focus on biomarkers of the vitreous antioxidant state in DR, AMD, RD, and PVR. These biomarkers are potential hallmarks to evaluate vitreoretinal disease progression and to provide new and innovative therapeutic targets for vitreoretinal disorders. Proteins found to be differentially expressed in these studies were used to perform functional enrichment analyses in STRING v11.5 to gather new insights into the role of ROS in these diseases and to find potential markers for the detection of early stages of OS, essential to prevent further retinal harmful effects. Additionally, several molecules were described as antioxidants with therapeutic potential in preclinical studies. Although the results of clinical trials are inconclusive to date, they offer great hope for the future development of new therapeutic strategies based on antioxidative treatments.

## Figures and Tables

**Table 1:** Summary of antioxidants, molecules essential for the activity of some antioxidant enzymes and enzymatic systems for reducing oxidative stress (OS).

Metabolite/Compound	Role	Structure	Reference
Ascorbic acid	Ascorbic acid, also called ascorbate or vitamin C, is a redox (reduction-oxidation) catalyst.	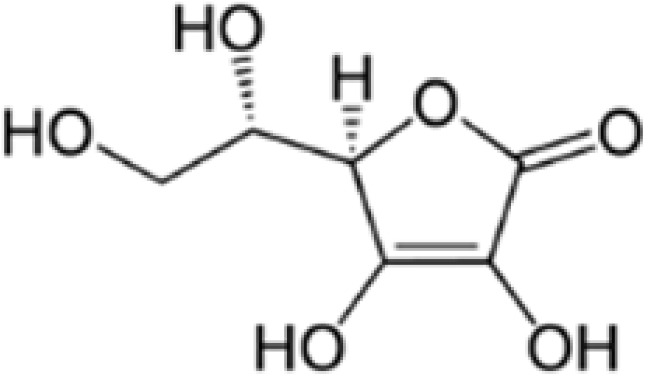	[268]
Lipoic acid	Responsible for stimulating the biosynthesis of GPX, an enzyme that has a significant free radical neutralizing effect. GPX neutralizes one of the most aggressive free radicals for the skin, the peroxide radical, transforming it into water.	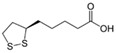	[269]
Uric acid	Acts as an antioxidant by mitigating OS caused by hypoxic.	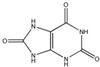	[270]
Carotenes	Antioxidant agents. β-Carotene prevent night blindness.	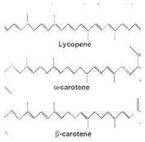	[271]
Glutatione	Water-soluble antioxidant, recognized as a non-protein thiol. It can be found in a reduced (GSH) or oxidized form (GSSG, dimerized form of GSH). The GSH/GSSG ratio is commonly used to estimate the redox state of biological systems.	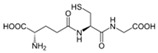	[272]
Tocopherols and tocotrienols (vitamin E)	The α-tocopherol form is the most important lipid-soluble antioxidant. It protects membranes from oxidation by reacting with lipid radicals produced in the reactive chain of lipid peroxidation. This eliminates the intermediate free radicals and prevents the spreading reaction from continuing.	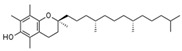	[273]
Ubiquinol or Coenzyme Q	Benzoquinone present in all the cells of the organism that participates in the processes of ATP production. It works as an antioxidant.	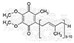	[274]
Transition Metal Chelates	Prevent catalyzing the production of free radicals in the cell, e.g., iron in the protein ferritin.	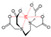	[275]
Selenium	It has no antioxidant action on its own but it is required for the activity of some antioxidant enzymes. It plays an important role in antioxidant selenoproteins to protect against OS initiated by excess ROS and NOS.	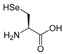 Selenium cysteine	[276]
Zinc	Ability to slow down oxidative processes. Zinc induces the synthesis of metallothioneins, which are proteins effective in reducing OH^−^ radicals and sequestering ROS produced in stressful situations, such as in type 2 diabetes.	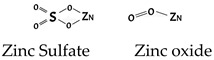	[277]
Melatonin	A powerful antioxidant, acting in the recovery of epithelial cells exposed to ultraviolet radiation and, through supplemental administration.	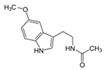	[278]
Enzyme systems	Cells are protected from OS by an interactive network of antioxidant enzymes. Through these enzyme networks, ROS released in essential metabolic processes such as oxidative phosphorylation are initially converted to H_2_O_2_ and later reduced to water. This detoxification pathway is composed of multiple enzymes.	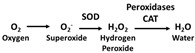 Enzyme pathway for the detoxification of reactive oxygen species.	[261]
Superoxide dismutase (SOD)	Class of structurally related enzymes that catalyze the hydrolysis of O_2_^−^ into oxygen and H_2_O_2_. SOD catalyzes the dismutation of superoxide into oxygen and H_2_O_2_. Because of this, it is an important antioxidant defense in most cells exposed to oxygen.	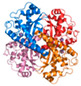	[279]
Catalases (CAT)	Enzymes that catalyze the conversion of H_2_O_2_ into water and oxygen, using iron or manganese as a cofactor.	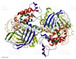	[280]
Peroxiredoxins (PRDX)	Peroxidases that catalyze the reduction of H_2_O_2_, organic hydroperoxides, and peroxynitrite.	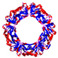 AhpC, a perirredoxin	[281]
Thioredoxin system	Contains the protein thioredoxin and its partner thioredoxin reductase. Thioredoxin operates as an effective reducing agent, eliminating ROS and keeping other proteins in their reduced state.	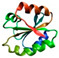	[282]
Glutathione system	Comprises glutathione, glutathione reductase, glutathione peroxidases, and GSTP1. Glutathione peroxidase is an enzyme-containing four selenium cofactors that catalyze the separation of H_2_O_2_ and organic hyperoxides.	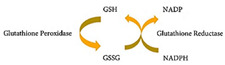	[283]

**Table 2:** Summary of the therapeutic strategies against oxidative stress (OS) and their use for certain diseases, including diabetes and its complications.

Drug	Disease	Action	NTC/Study Phase	References
Intravitreal Corticosteroids/triamcinolone acetonide	DR	Triamcinolone acetonide inhibits NF-κB and MAPK pathways, RAAS blockers, and PKC inhibitors. Intravitreal triamcinolone inhibits the p38 MAPK pathway, exerting neural protective effects on retinal neurons in diabetes.	Not applicable	[292]
GC4419 (AVASOPASEM)	Oral squamous cell carcinoma	GC4419 is an antioxidant that is considered a SOD mimic. It has been studied in a phase I dose-escalation study of GC4419 in combination with radiation and chemotherapy for squamous cell cancers of the head and neck.	NCT01921426/Phase I	[293]
XXS	Hyperlipidemia	XXS (a mixture of natural polyphenolic extracts of edible plants) has a significant and favourable effect on OS notably with a decrease in certain markers and on plasma lipid parameters. It was studied in a randomized, double-blind study to evaluate the effects of XXS on OS in patients with mild or moderate hyperlipidemia and on lipoprotein kinetics.	NCT02826083/Not applicable	[294]
AT-001	OS	Studied in a phase I clinical trial named: “Multiple-ascending dose clinical trial of the safety and tolerability of antioxidant (AT-001) treatment for reducing brain OS“. The purpose of this study was to determine the safety, bioavailability, and effectiveness of an organic yeast-selenium compound in reducing brain OS.	NCT01731093/Phase I	[295]
N-acetyl cysteine/omega 6 Fish oil (PUFA)	Ameliorating OS in type 1 DM	It has been studied in an active ongoing early phase 1 study called: “Supplementation of N-acetylcysteine and arachnoid acid in type 1 DM to determine changes in OS”.	NCT03056014/Early Phase I	[296]
Lutein	OS in healthy subjects	Two doses of lutein 20 and 10 mg versus placebo were studied in this clinical trial to examine the effect of consuming different doses of lutein on OS in healthy non-smoker subjects.	NCT01056094/Phase I/Phase II	[297]
Oxytocin nasal spray	OS and inflammation	This study evaluated the potential benefits of intranasal oxytocin on undersea Operator training and performance: hyperoxic swim-Induced OS and inflammation	NCT04732247/Phase II	[298]
Calcined magnesia/Ezetimibe/simvastatin/Rosuvastatin	OS and diabetic polyneuropathy	This randomized, double-blinded, placebo-controlled clinical trial evaluated the effect of ezetimibe/simvastatin and rosuvastatin on OS and mitochondrial function in patients with Diabetic Polyneuropathy.	NCT02129231/Phase II	[299,300]
Galvus (vildagliptin)/pioglitazone	DM/OS	This study compared the effect of vildagliptin vs. pioglitazone to OS on daily blood glucose fluctuations, in patients with type 2 DM that were inadequately controlled by metformin.	NCT01339143/Phase IV	[301]
Pterostilbene	Hyperlipidemia/blood pressure/OS	Pterostilbene is one of several stilbenes found in certain berries, particularly blueberries, that have demonstrated pre-clinical benefit to cholesterol, blood pressure, and OS. The purpose of this study was to evaluate whether pterostilbene will help control cholesterol and blood pressure, as well as improve markers for OS in patients with dyslipidemia.	NCT01267227/Phase II/Phase III	[302]
Combined antioxidant therapy: lutein + astaxanthin + zeaxanthin + vitamin C + vitamin E + zinc + copper (Drusen Laz)	DR/OS/DM	This clinical trial aimed at evaluating the effect of combined antioxidant therapy on the levels of OS markers in the aqueous and vitreous humour of patients with PDR.	NCT04071977/Phase II	[303]
IMMUSYSTEM	OS	This study evaluated the antioxidant and anti-inflammatory capacity of nutraceutical immusystem food supplement (evaanis) to verify the effectiveness of nutraceutical immu·system dietary supplement in reducing the levels of OS and inflammation in a sample of healthy adult subjects with high baseline levels of OS.	NCT04912947/Not Applicable	[304]
Xanthohumol	OS	The purpose of this research study was to determine if xanthohumol prevents damage to DNA and OS.	NCT02432651/Phase I	[305]
MITO-AO: the mitochondrial-targeted antioxidant (MITO-AO) mitoquinone PB-125: a novel naturally occurring Nrf2 activator	Aging/OS/vascular endothelium/skeletal muscle/antioxidants	This clinical trial is studying the targeting OS to prevent vascular and skeletal muscle dysfunction during disuse. It has two aims. In the first aim, the mitochondrial-targeted antioxidant (MITO-AO) mitoquinone was administered during disuse to improve free radical scavenging at the level of the mitochondria. In the second aim, activation of Nuclear Factor Erythroid-2-like 2 (Nrf2) the “master regulator of antioxidant enzymes” was accomplished with PB125 (a novel naturally occurring Nrf2 activator) to augment endogenous antioxidant defense systems.	NCT04351113/Not Applicable	[286]
N-Acetyl cysteine/Proimmune 200/FT061452	OS	N-Acetyl Cysteine is used as a dietary supplement and it has been reported to increase glutathione levels in the body. The diet supplement called ProImmune is also changed by the body into glutathione. In this clinical trial named PILOT it was studied the effects of short-term administration of a novel glutathione precursor (ft061452), on serum and intracellular glutathione levels.	NCT01251315/Phase I	[306]
Lactobacillus rhamnosus Lactobacillus casei Bifidobacterium longum	OS	This clinical study determined the efficacy of the investigational products (*Lactobacillus rhamnosus* and *Lactobacillus casei* and *Bifidobacterium longum*) versus placebo (maltodextrin and sucrose) in reducing OS during the performance of a physical exercise of a certain intensity and duration.	NCT03798821/Not Applicable	[307,308]
Metadoxine	NAFLD (Non-alcoholic fatty liver disease)/pre-diabetes	This study was performed to investigate the effect of metadoxine on OS in non-alcoholic fatty liver disease prediabetic Mexican patients. Investigators proposed that metadoxine is a possible modifier of the OS in non-alcoholic liver disease, prediabetic patients.	NCT02051842/Phase IV	[309]
Vildagliptin Glimepiride	Type 2 DM	This clinical was performed to evaluate the effect of vildagliptin-based treatment versus sulfonylurea on glycemic variability, OS, glp-1, and endothelial function in patients with type 2 DM.	NCT01404676/Phase IV	[310]
Controlled-release oral alpha-lipoic acid	Type 1 DM	It has been hypothesized that alpha-lipoic acid, a potent antioxidant, can stop ROS from forming, thereby preventing long-term complications in DM. Therefore it was conducted a pilot study on the effect of oral controlled-release alpha-lipoic acid on OS in type 1 DM adolescents.	NCT00187564/Not Applicable	[311]
Sitagliptin Glimepiride	Type 2 DM	This research focused on the effect of the dipeptidyl peptidase-iv inhibitor Sitagliptin on 24 h glycemic excursion and improvement of OS markers compared to long-acting sulphonyl urea Glimepiride in type 2 DM patients with inadequate glycemic control on metformin.	NCT00699322/Phase IV	[312]
Vildagliptin	Microvascular function/OS/inflammation	The purpose of this study was to determine whether vildagliptin, evaluated in obese and diabetic women, has vascular protective effects and whether the regulatory mechanisms of these actions correlate with OS, inflammatory markers, and intestinal peptides in baseline state and after a lipid overload.	NCT01827280/Phase IV	[313,314]
Linagliptin	Type 2 DM	This clinical trial investigated the effect of TRADJENTA^®^ (linagliptin) on inflammation, OS and insulin resistance in obese type 2 DM subjects.	NCT02372630/Phase IV	[315]
Ubiquinol	OS/inflammation/muscle injury	Ubiquinol is a well-known antioxidant. This study was conducted to investigate the influence of short-term supplementation with ubiquinol on diverse aspects related to physical activity (muscle function, OS, and inflammatory signalling).	NCT01940627/Phase II/Phase III	[316]
Simvastatin	OS	Statins (atorvastatin, simvastatin, pravastatin, and rosuvastatin) are the drugs that have antioxidant properties. The clinical trial SIMOX-Induction of OS (SIMOX) was a randomized, double-blinded, placebo-controlled study of simvastatin’ possible effect on OS on healthy volunteers. The purpose of the study was to investigate if the use of simvastatin is associated with the level of OS in humans.	NCT02256254/Phase II	[317]
Propofol	OS	Propofol, a highly liposoluble anesthetic, has been shown in vitro and in vivo to have a significant antioxidant effect against lipid peroxidation. In humans, propofol reduces ischemia-reperfusion-induced lipid peroxidation. This clinical study was performed to demonstrate that propofol may protect against gut hypoperfusion-reperfusion injury during robot-assisted laparoscopic radical prostatectomy.	NCT01334424/Not applicable	[318]

Abbreviations: Diabetes mellitus (DM); diabetic retinopathy (DR); Oxidative stress (OS); proliferative diabetic retinopathy (PDR).

## Supplementary Materials

The following supporting information can be downloaded at https://www.mdpi.com/article/10.3390/antiox11030505/s1, Table S1: List of proteins found differently expressed in DR vitreous and functional analysis; Table S2: List of proteins found differently expressed in AMD vitreous and functional analysis; Table S3: List of proteins found differently expressed in RRD/PVR vitreous and functional analysis.

## Abbreviations

ACE	Angiotensin-Converting Enzyme
AGEs	Advanced glycation end products
AGT	Angiotensinogen
AKR1A1	Aldo-Keto Reductase Family 1 Member A1
AMD	Age-Related Macular Degeneration
Ang II	Angiotensin II
APOE	Apolipoprotein E
AT1R	Angiotensin II type 1 receptor
AT2R	Angiotensin II type 2 receptor
Aβ	Beta Amyloid
BRB	Blood–retinal barrier
CAT	Catalase
CLU	Clusterin
CMA	Chaperone-Mediated Autophagy
CNV	Choroidal Neovascularization
DR	Diabetic Retinopathy
EMT	Epithelial–Mesenchymal Transition
eNOS	Endothelial Nitric Oxide Synthase
ERM	Epiretinal membranes
GPX	Glutathione Peroxidase
GSH	Reduced Glutathione
GSTP1	Glutathione S-Transferase
H_2_O_2_	Hydrogen Peroxide
HP	Haptoglobin
HSPs	Heat Shock Proteins
ICAM1	Intercellular Adhesion Molecule 1
IDH1	Isocitrate dehydrogenase [NADP] cytoplasmic
IL1β	Interleukin-1 beta
iNOS	Inducible Nitric Oxide Synthase
JAK-STAT	Janus Kinase-Signal Transducer and Activator of Transcription
KEGG	Kyoto Encyclopedia of Genes and Genomes
hsa	Kyoto Encyclopedia of Genes and Genomes Entry
LAMP2	Lysosome-associated membrane glycoprotein 2
MH	Macular hole
MMP8	Neutrophil Collagenase
mTOR	Mammalian Target of Rapamycin
nAMD	“Wet” or Neovascular Age-Related Macular Degeneration
NF-κB	Nuclear Factor Kappa B
nNOS	Neuronal Nitric Oxide Synthase
NO	Nitric Oxide
NOS	Nitric Oxide Synthase
NOX	NADPH oxidase
Nrf2	Nuclear Factor Erythroid 2-Related Factor 2
O_2_^−^	Superoxide
OH^−^	Hydroxyl
ONOO−	Peroxynitrite
OS	Oxidative Stress
PARK7	Parkinson Disease Protein 7
PDR	Proliferative Diabetic Retinopathy
PEDF	Pigment Epithelium-Derived Factor
PI3K-Akt	Phosphatidylinositol 3-Kinase/Protein Kinase B
PKC	Protein Kinase C
PRDX	Peroxiredoxin
PRDX1	Peroxiredoxin-1
PRDX2	Peroxiredoxin-2
PRDX5	Peroxiredoxin-5
PRDX6	Peroxiredoxin-6
PUFAs	Polyunsaturated Fatty Acids
R-HSA	Reactome Entry
PVR	Proliferative Vitreoretinopathy
RAGE	Receptor for advanced glycation end products
RAS	Renin–Angiotensin System
RD	Retinal Detachment
RNS	Reactive Nitrogen Species
ROS	Reactive Oxygen Species
RPE	Retinal Pigment Epithelium
RRD	Rhegmatogenous Retinal Detachment
SOD	Superoxide Dismutase
SOD1	Superoxide Dismutase [Cu-Zn]
SOD3	Extracellular Superoxide Dismutase [Cu-Zn]
TICAM1	TIR Domain-Containing Adapter Molecule 1
TNF-α	Tumor Necrosis Factor
VCAM1	Vascular Cell Adhesion Protein 1
VEGF	Vascular endothelial growth factor
WHO	World Health Organization
